# Neurophysiological tests in the neuro ICU

**DOI:** 10.1186/cc14540

**Published:** 2015-03-16

**Authors:** A Marudi, Y Valzani, F Valzania, M Carpentiero, C Parenti, S Scacchetti, S Baroni, E Bertellini

**Affiliations:** 1 Nuovo Ospedale Civile Sant'Agostino Estense, Modena, Italy; 2 University of Modena and Reggio Emilia, Modena, Italy

## Introduction

Neurophysiological tests (NPTs) are important prognostic and diagnostic tools for patients admitted to the modern neuro ICU (NICU) [1]. Electroencephalography (EEG), somasensorial evoked potentials (SSEP), auditory brainstem response (ABR) and electromyography (EMG) complete clinical examination and radiological findings in patients suffering from post-traumatic brain injury, post-anoxic brain injury, refractory male epiletticus status, and neuromuscular illness. We evaluate the spread of NPTs in our NICU.

## Methods

We collected data from patients admitted to our NICU from January 2014 to November 2014. We recorded the admission diagnosis and the NPT applied.

## Results

From January 2014 to November 2014 we performed 521 EEG, 45 SSEP/ABR and 10 EMG. In post-anoxic and post-traumatic brain-injured comatose patients we performed EEG, SSEP and ABR 24 to 48 hours after the admission to predict later prognosis and expected neurological deficit [2]. In the presence of a benign pattern no further evaluation was performed; in the presence of a malignant pattern the NPTs were repeated every 48 to 72 hours according to the protocol of our institute. In post-anoxic comatose patients we recorded EEG during hypothermia to assure burst suppression. In post-traumatic brain-injured patients with a persistent comatose state we use EEG to detect nonconvulsive states which potentially can increase secondary brain injury if untreated. In malignant epilepticus status we use EEG to monitor the effect of therapy and to modify it. In patients who present profound weakness of legs and hands we performed EMG to distinguish primary peripheral myopolyneuropathy (Guillian Barrè, miastenia gravis) from secondary illness acquired in the ICU (critical polyneuropathy, critical myopathy) [3].

## Conclusion

NPT can improve management of patients admitted to the neuro ICU. The data provided can modify therapeutic strategies and improve outcome in these settings of patients.
